# Short-Term Clinical and Immunologic Outcomes Conditional on SVR12 in Hepatitis C Virus-Associated Cryoglobulinemic Vasculitis Treated With Direct-Acting Antivirals: A Systematic Review and Meta-Analysis

**DOI:** 10.7759/cureus.103349

**Published:** 2026-02-10

**Authors:** Boonphiphop Boonpheng, Patompong Ungprasert

**Affiliations:** 1 Division of Nephrology, Department of Internal Medicine, University Hospitals Cleveland Medical Center, Cleveland, USA; 2 Medicine, Case Western Reserve University School of Medicine, Cleveland, USA; 3 Department of Rheumatic and Immunologic Diseases, Cleveland Clinic, Cleveland, USA

**Keywords:** direct acting antiviral (daa), hepatitis c (hcv) infection, hepatitis c virus-associated cryoglobulinemic vasculitis, mixed cryoglobulinemic vasculitis, sustained virologic response (svr)

## Abstract

In contemporary clinical practice, direct-acting antiviral therapy (DAA) produces high rates of sustained virologic response (SVR) among patients with hepatitis C virus infection; however, short-term clinical and immunologic outcomes following viral eradication in HCV-associated cryoglobulinemic vasculitis remain incompletely characterized, particularly among patients who have already achieved SVR12. A systematic review and meta-analysis was performed to evaluate short-term outcomes conditional on achieving SVR12 among patients with symptomatic HCV-associated cryoglobulinemic vasculitis treated with interferon-free DAA therapy. EMBASE and PubMed were searched from inception through January 2026, and two reviewers independently screened studies and extracted data. Pooled proportions of complete clinical response, partial clinical response, and complete immunologic response at the time of SVR12 were calculated using random-effects models. Eighteen observational studies were included. Among patients who achieved SVR12, the pooled proportion of complete clinical response was 64.8% (95% CI: 49.6%-79.3%), while partial clinical response occurred in 23.8% (95% CI: 18.3%-30.4%). Complete immunologic response was observed in 43.9% of patients (95% CI: 36.6%-51.5%). Substantial heterogeneity was observed for clinical outcomes reflecting differences across observational cohorts, whereas heterogeneity was lower for immunologic response. In conclusion, among patients with HCV-associated cryoglobulinemic vasculitis who achieved SVR12 with DAA therapy, short-term clinical improvement was common, although complete immunologic normalization was less frequent. These findings provide clinically relevant prognostic information regarding early expectations following viral eradication, while highlighting that longer-term durability of response requires ongoing evaluation.

## Introduction and background

Among recognized etiologies, hepatitis C virus infection accounts for the majority of cases of mixed cryoglobulinemic vasculitis, an immune complex-mediated small- and medium-vessel vasculitis with heterogeneous clinical manifestations, including purpura, arthralgia, peripheral neuropathy, and glomerulonephritis. The pathogenesis of HCV-associated cryoglobulinemic vasculitis is closely linked to chronic viral antigenic stimulation, resulting in B-cell activation and clonal expansion, production of circulating cryoglobulins, complement consumption, and subsequent vascular inflammation [1-3].

In contemporary clinical practice, interferon-free direct-acting antiviral therapy achieves high rates of sustained virologic response (SVR) in patients with chronic hepatitis C infection, including those with extrahepatic manifestations [4-6]. Viral eradication is believed to remove the primary antigenic driver of cryoglobulin production and vasculitic activity, and multiple studies have reported improvement or resolution of cryoglobulinemic vasculitis following achievement of SVR [4-8]. However, clinical experience in the direct-acting antiviral therapy (DAA) era has demonstrated substantial heterogeneity in response. While many patients experience early symptomatic improvement, others exhibit partial clinical response, persistent immunologic abnormalities, or relapses despite confirmed viral clearance [9-12].

Interpretation of treatment outcomes in HCV-associated cryoglobulinemic vasculitis is further complicated by marked variability across published studies. Included cohorts differ widely with respect to disease severity, organ involvement, and use of concomitant immunosuppressive therapies. In addition, definitions of clinical and immunologic response are inconsistent, ranging from symptom-based assessments to organ-specific or score-based measures such as the Birmingham Vasculitis Activity Score. These factors limit the ability to directly compare results across studies and contribute to uncertainty regarding expected outcomes following viral eradication [13,14].

Moreover, several longitudinal cohorts with extended follow-up have demonstrated that persistence or recurrence of cryoglobulinemic vasculitis may occur months to years after achievement of SVR, even in the setting of sustained viral suppression. Studies from French and Italian cohorts have shown that immunologic abnormalities, clonal B-cell populations, and clinical disease activity may persist or re-emerge despite successful antiviral therapy, highlighting a potential dissociation between viral eradication and durable immune remission [9,15-17].

Given these observations, an important and clinically relevant question remains incompletely addressed. That is, among patients with symptomatic HCV-associated cryoglobulinemic vasculitis who have achieved SVR12 with DAA therapy, what degree of clinical and immunologic response can be expected in the short term? Addressing this conditional, post-eradication question is distinct from evaluating antiviral treatment effectiveness and provides prognostic information relevant to early post-SVR management.

Accordingly, we conducted a systematic review and meta-analysis to evaluate short-term clinical and immunologic outcomes conditional on achieving SVR12 among patients with symptomatic HCV-associated cryoglobulinemic vasculitis treated with DAA therapy. Specifically, we aimed to estimate pooled rates of complete clinical response, partial clinical response, and complete immunologic response at the time of SVR12.

## Review

Materials and methods

Search Strategy and Data Sources

A systematic literature search was conducted using the EMBASE and PubMed databases from inception through January 2026. The search strategy combined controlled vocabulary terms and free-text keywords related to hepatitis C virus infection, cryoglobulinemia or cryoglobulinemic vasculitis, direct-acting antiviral therapy, and sustained virologic response. Detailed database-specific search strategies are provided in the Appendix. Reference lists of included studies were also screened to identify additional relevant publications.

Study Selection

Two investigators (BB and PU) independently evaluated titles and abstracts to determine eligibility. Full-text review was subsequently performed for studies deemed potentially relevant by either reviewer. Any disagreements regarding study inclusion were resolved through reviewer discussion.

Eligibility for inclusion required fulfillment of the following criteria: (1) included adult patients with symptomatic HCV-associated cryoglobulinemic vasculitis; (2) evaluated treatment with interferon-free direct-acting antiviral therapy; (3) reported outcomes among patients who achieved sustained virologic response at 12 weeks (SVR12); (4) reported clinical and/or immunologic response outcoms assessed at or near the time of SVR12.

Studies were excluded if they met any of the following criteria: (1) included only asymptomatic cryoglobulinemia; (2) were conducted exclusively in the interferon-based treatment era; (3) included mixed HCV monoinfected and HIV/HCV/HBV coinfected populations without separable outcome reporting; (4) reported outcomes at time points other than SVR12 (e.g., SVR24 or SVR48 only); (5) lacked separate reporting of complete and partial clinical response outcomes.

A formal risk-of-bias assessment was not undertaken, as most included studies were observational cohorts with consecutive enrollment and insufficient reporting granularity to support standardized bias assessment tools.

Data Extraction

Study data were independently extracted by two reviewers using a standardized data collection form. Extracted variables included study characteristics, country, study design, sample size of patients achieving SVR12, predominant clinical manifestations, presence of renal involvement, antiviral regimen, use of concomitant immunosuppressive therapy, and duration of follow-up to SVR12. Any disagreements regarding study inclusion were resolved through reviewer discussion.

Outcomes and Definitions

The primary outcomes of interest were pooled proportions of: (1) complete clinical response; (2) partial clinical response; (3) complete immunologic response. All were assessed at the time of SVR12 among patients who achieved SVR12.

Definitions of clinical and immunologic response were accepted as reported by individual studies and are summarized in Table 2. Given substantial heterogeneity in how clinical and immunologic responses were defined across cohorts, outcomes were pooled as reported rather than standardized post hoc. Complete clinical response was most commonly defined as resolution or improvement of all baseline vasculitic manifestations or affected organs, although several studies incorporated validated disease activity scores, such as the Birmingham Vasculitis Activity Score. Partial clinical response generally reflected incomplete but meaningful improvement in clinical manifestations. Complete immunologic response was most often defined by the disappearance of detectable cryoglobulins, with or without normalization of complement levels or rheumatoid factor. No attempt was made to retrospectively standardize outcome definitions across studies.

Statistical Analysis

We conducted statistical analyses using Comprehensive Meta-Analysis (CMA) software (Biostat, Englewood, NJ). The analytic framework was designed to estimate outcome probabilities conditional on achieving SVR12, rather than intention-to-treat treatment effectiveness. Pooled proportions were estimated using random-effects models, given anticipated clinical and methodological heterogeneity across studies. Results are reported with corresponding 95% confidence intervals (CI). Between-study variability was quantified using the I² statistic.

Reporting Standards

**Figure 1:**
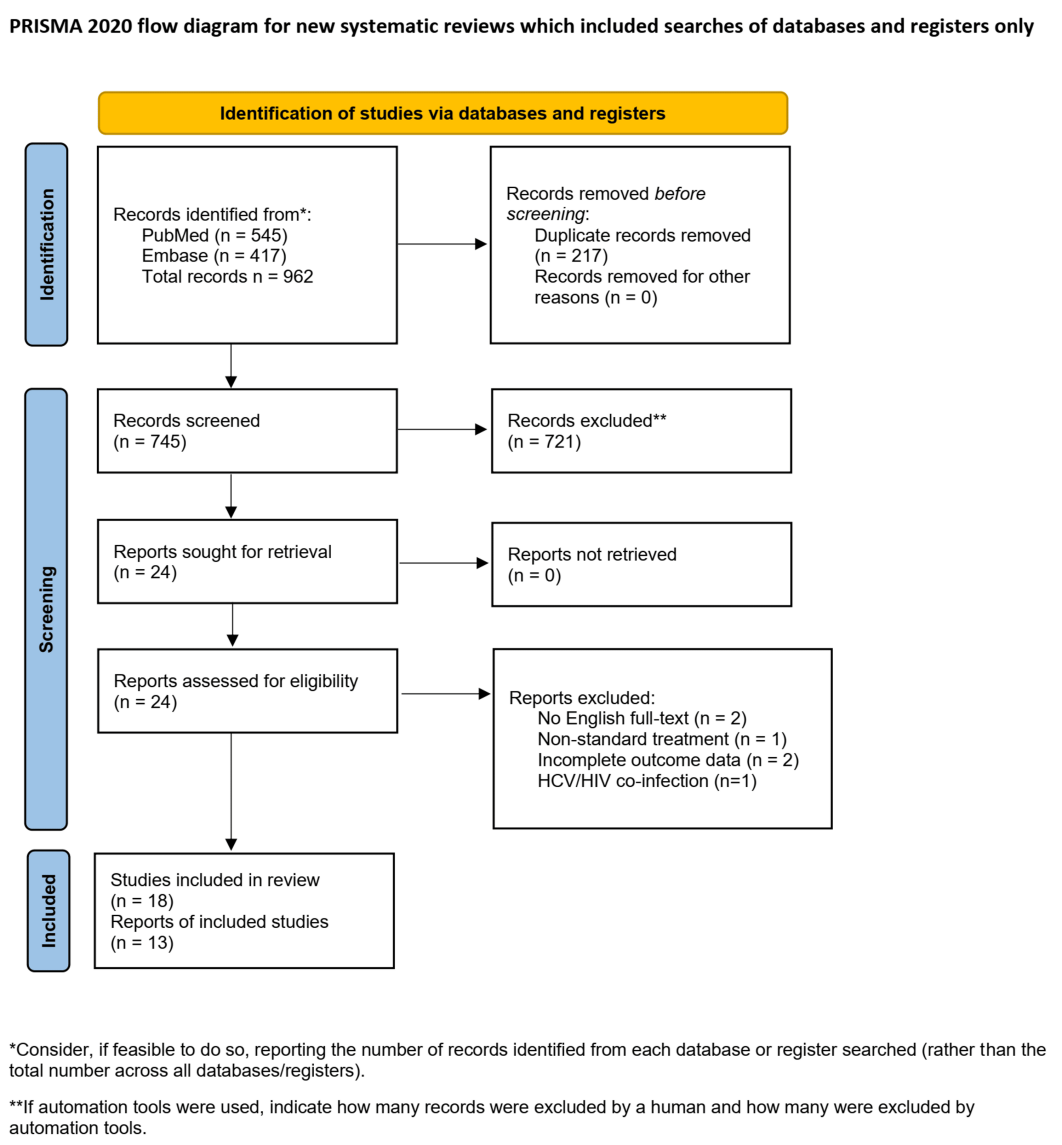
PRISMA flow diagram of study selection PRISMA: Preferred Reporting Items for Systematic Reviews and Meta-Analyses

We conducted this systematic review and meta-analysis in accordance with established methodological standards for observational meta-analyses. Study selection is summarized using a Preferred Reporting Items for Systematic Reviews and Meta-Analyses (PRISMA) flow diagram, as detailed in Figure 1.

Results

Study Selection

Using EMBASE and PubMed with the predefined search strategy, 256 records were identified through January 2026. Following title and abstract screening, potentially eligible studies underwent full-text review. Six studies were excluded at the full-text stage for the following reasons: full text published in Russian with insufficient abstract data (n = 2), antiviral therapy duration limited to four weeks with outcomes reported at SVR48 (n = 1), conference poster abstract without separate reporting of complete and partial clinical response (n = 1), inclusion of mixed HIV/HCV coinfected populations without separable outcome reporting (n = 1), and incompatible outcome timing (n = 1). Following additional manual reference list screening, a total of 13 studies were included in the final meta-analysis [4,6-8,16,18-24].

All reported outcome estimates reflect probabilities among patients who achieved SVR12.

Study Characteristics

An overview of study characteristics is provided in Table 1, all of which were observational in nature. Sample sizes varied widely, ranging from small single-center cohorts consisting of patients with more severe disease manifestations to large multicenter cohorts dominated by cutaneous and constitutional symptoms. The proportion of patients with renal involvement varied substantially across studies. A range of sofosbuvir- and protease inhibitor-based DAA regimens were used, reflecting evolving treatment eras and viral genotypes.

**Table 1:** Characteristics of included studies DAA, direct-acting antivirals; HCV-CV, Hepatitis C virus-associated cryoglobulinemic vasculitis; N/A, not available; PegIFN, pegylated-interferon; SVR, sustained virologic response

Study	Year	Country	Design	Sample Size (HCV-CV)	Clinical manifestations	DAA regimen	Concomitant Immunosuppression	Follow-Up
Sise et al. [4]	2015	United States	Retrospective case series	12 (10 achieved SVR)	Glomerulonephritis 58%, Purpura 50%, Arthralgia 50%, Peripheral neuropathy 33%, Raynaud’s phenomenon 17%, Sicca 8%, Renal arteritis/infarct 8%	Sofosbuvir + ribavirin; sofosbuvir+ simeprevir	Combinations of rituximab, corticosteroids, and plasmapheresis 6/7 prior to DAA therapy; only 1 was receiving ongoing immunosuppression concurrent with DAA	At least 24 weeks
Gragnani et al. [6]	2016	Italy	Prospective cohort	44 (100% SVR)	Palpable purpura (73%), arthralgia (59%), weakness (77%), peripheral neuropathy (63%), Raynaud’s phenomenon (32%), renal involvement (9%), sicca syndrome (41%), and skin ulcers (14%)	Sofosbuvir + ribavirin; sofosbuvir + simeprevir (+ ribavirin); sofosbuvir + daclatasvir (+ ribavirin); sofosbuvir + ledipasvir (+ ribavirin)	2 with severe vasculitis received rituximab concurrently with DAA therapy	42 weeks
Emery et al. [7]	2017	Canada	Prospective cohort	18 (88.9% SVR)	Dermatological 83.3%, Neurological 27.7%, Renal 55.5%, Musculoskeletal 16.7%	pegIFN + ribavirin + telaprevir; pegIFN + ribavirin + boceprevir; pegIFN + ribarivin + sofosbuvir; sofosbuvir + ribarivin; sofosbuvir + simeprevir; sofosbuvir + ledipasvir +/- ribavirin; paritaprevir/ritonavir + ombitasvir + dasabuvir +/- ribavirin	4 plasmaphereses, 3 rituximab	48 weeks
Bonacci et al. [8]	2017	Spain	Prospective cohort	35 with symptomatic crryoglobulinemic vasculitis (94% SVR)	Purpura 65%, Arthralgia 31%, Weakness 70%, Polyneuropathy 50%, Renal involvement 20%	Ombitasvir/paritaprevir/ritonavir + dasabuvirl; sofosbuvir + ledipasvir; sofosbuvir+ simeprevir; simeprevir + daclatasvir + sofosbuvir; sofosbuvir+ daclatasvir; PegIFN/DAAs	Corticosteroids 37%, Rituximab 8%	At least 24 weeks
Bonacci et al. [10]	2018	Spain	Prospective cohort	42 (100% SVR12)	Purpura (63%), Weakness (61%), Neuropathy (41%), and Nephropathy (20%)	N/A	Corticosteroids were reduced or discontinued, and 3 received rituximab	24 weeks
Kondili et al. [16]	2021	Italy	Prospective multicenter cohort	423	Purpura, Asthenia, and Arthralgia > Neuropathy and Sicca > Renal involvement 12.2%	N/A	Rituximab (% not specified)	3 months
Solima et al. [18]	2016	Italy	Case series	7 (100% SVR)	Arthralgias, Purpura, Peripheral Neuropathy, Skin ulcers	Ombitasvir/paritaprevir/ritonavir + dasabuvir; sofosbuvir + ribavirin; sofosbuvir + daclatasvir; sofosbuvir + simeprevir	None	Up to 48 weeks
Comarmaud et al. [19]	2017	France	Prospective cohort	27	Active HCV vasculitis is defined by clinically active vasculitis with skin, joint, renal, peripheral nerve, central neurologic, digestive, pulmonary, and/or cardiac involvement	Sofosbuvir + ribavirin; sofosbuvir + daclatasvir; sofosbuvir + simeprevir	None	At least 24 weeks
Laulette et al. [20]	2017	Italy	Prospective cohort	22	Meltzer’s triad 100%, Glomerulonephritis 18.1%, Peripheral neuropathy 9.1%	Ofosbuvir + ribavirin, ombitasvir/paritaprevir/ritonavir plus dasabuvir, +/- ribavirin; sofosbuvir + ledipasvir; sofosbuvir + daclatasvir	N/A	At least 24 weeks
Saadoun et al. [21]	2017	France	Prospective multicenter cohort	41	Purpura (75.6%), Arthralgia (63.4%), Peripheral neuropathy (51.2%), Skin ulcers (17.1%), Glomerulonephritis (12.2%), Gut involvement and myocarditis (2.4%)	Sofosbuvir + daclatasvir	2 received rituximab; 3 received plasmapheresis	26 months
Hassan et al. [22]	2018	Egypt	Prospective cohort	120	Glomerulonephritis 11%, Meltzer’s triad 100%, Purpura 100%, Peripheral neuropathy 52.3%	Sofosbuvir + daclatasvir	N/A	At least 24 weeks
Cacoub et al. [23]	2019	Mutil-national	Prospective international multicenter cohort	148 (97.2% SVR)	Arthralgia 64.4%, Purpura 57.4%, Skin necrosis 10.1%, Neuropathy 58.1%, Renal involvement 16.9%, Hypertension 29.7%, Severe 29.1% (skin necrosis, glomerulonephritis, and involvement of the central nervous system, heart, or gut)	Sofosbuvir + daclatasvir; sofosbuvir + ribavirin; sofosbuvir + ledipasvir; sofosbuvir + simeprevir,	During antiviral therapy, 14.3% of patients received corticosteroids, immunosuppressants, or plasma exchange	15.3 months
Chang et al. [24]	2022	Taiwan, China	Prospective cohort	8	N/A	Multiple combinations	N/A	At least 24 weeks

Concomitant immunosuppressive therapy, including corticosteroids, rituximab, and plasma exchange, was frequently reported, although the timing and indication for immunosuppression varied across studies and were inconsistently described.

Outcome Definitions

Definitions of clinical and immunologic responses varied across included studies and are summarized in Table 2 and Table 3. Complete clinical response was most commonly defined as resolution or improvement of all baseline vasculitic manifestations or affected organs, although several studies incorporated validated disease activity scores, such as the Birmingham Vasculitis Activity Score. Partial clinical response was less consistently defined and generally reflected incomplete but clinically meaningful improvement. Definitions of complete immunologic response were more uniform and were most commonly based on the disappearance of detectable cryoglobulins, with or without normalization of complement levels or rheumatoid factor.

**Table 2:** Definitions of clinical response in each included study N/A, not available

Study	Complete Clinical Response	Partial Clinical Response
Sise et al. 2015 [4]	Resolution of all vasculitic manifestations	Improvement without complete resolution
Gragnani et al. 2016 [6]	Improvement of all the baseline symptoms	Disappearance or improvement of at least half of the baseline symptoms
Emery et al. 2017 [7]	All vasculitis symptoms needed to meet the criteria for resolution	Resolution of at least half of all baseline symptoms
Bonacci et al. 2017 [8]	Birmingham Vasculitis Activity Score (version 3) was 0, or if all affected organs improved 12 weeks after the end of therapy	Birmingham Vasculitis Activity Score (version 3), less than 50% of the baseline score or improvement in at least half of the involved organs from baseline
Bonacci et al. 2018 [10]	Birmingham Vasculitis Activity Score (version 3) of zero or by the improvement of all affected organs after antiviral therapy	Birmingham Vasculitis Activity Score (version 3) < 50% of the baseline score or improvement in at least half of the involved organs from baseline
Kondili et al. 2021 [16]	All baseline clinical manifestations had improved	An improvement in at least half of the baseline symptoms
Solima et al. 2016 [18]	N/A	N/A
Comarmond et al. 2017 [19]	Improvement of all the affected organs involved at baseline and the absence of clinical relapse	N/A
Lauletta et al. 2017 [20]	Regression of symptomatology (clinical response) and cryoglobulin disappearance or cryocrit reduction ≥50% (immunological response)	With or without either immunological or clinical response
Saadoun et al. 2017 [21]	Improvement of all the affected organs involved at baseline and no clinical relapse) after a median time of 12 weeks of therapy	N/A
Hassan et al. 2018 [22]	Clinical improvement of the presenting cryoglobulinemic-related manifestations and disappearance of cryoglobulins in serum	Either (1) Clinical improvement of the presenting cryoglobulinemic-related manifestations or (2) Disappearance of cryoglobulins in serum
Cacoub et al. 2019 [23]	Improvement of all organs involved at baseline and the absence of clinical relapse	Improvement in some but not all organs involved at baseline
Chang et al. 2022 [24]	Birmingham Vasculitis Activity Score (version 3) was 0, or if all affected organs improved 12 weeks after the end of therapy	N/A

**Table 3:** Definition of complete immunologic response in each included study RF, rheumatoid factors

Study	Complete Immunologic Response Definition
Gragnani et al. 2016 [6]	Disappearance of cryoglobulins
Emery et al. 2017 [7]	Disappearance of cryoglobulins
Bonacci et al. 2017 [8]	Circulating cryoglobulins became negative along with complement and/or RF normalization
Bonacci et al. 2018 [10]	circulating cryoglobulin test results became negative, along with complement and/or RF normalization
Comarmond et al. 2017 [19]	Disappearance of cryoglobulins
Lauletta et al. 2017 [20]	Disappearance of cryoglobulins
Cacoub et al. 2019 [23]	Disappearance of cryoglobulins

Complete Clinical Response at SVR12

**Figure 2:**
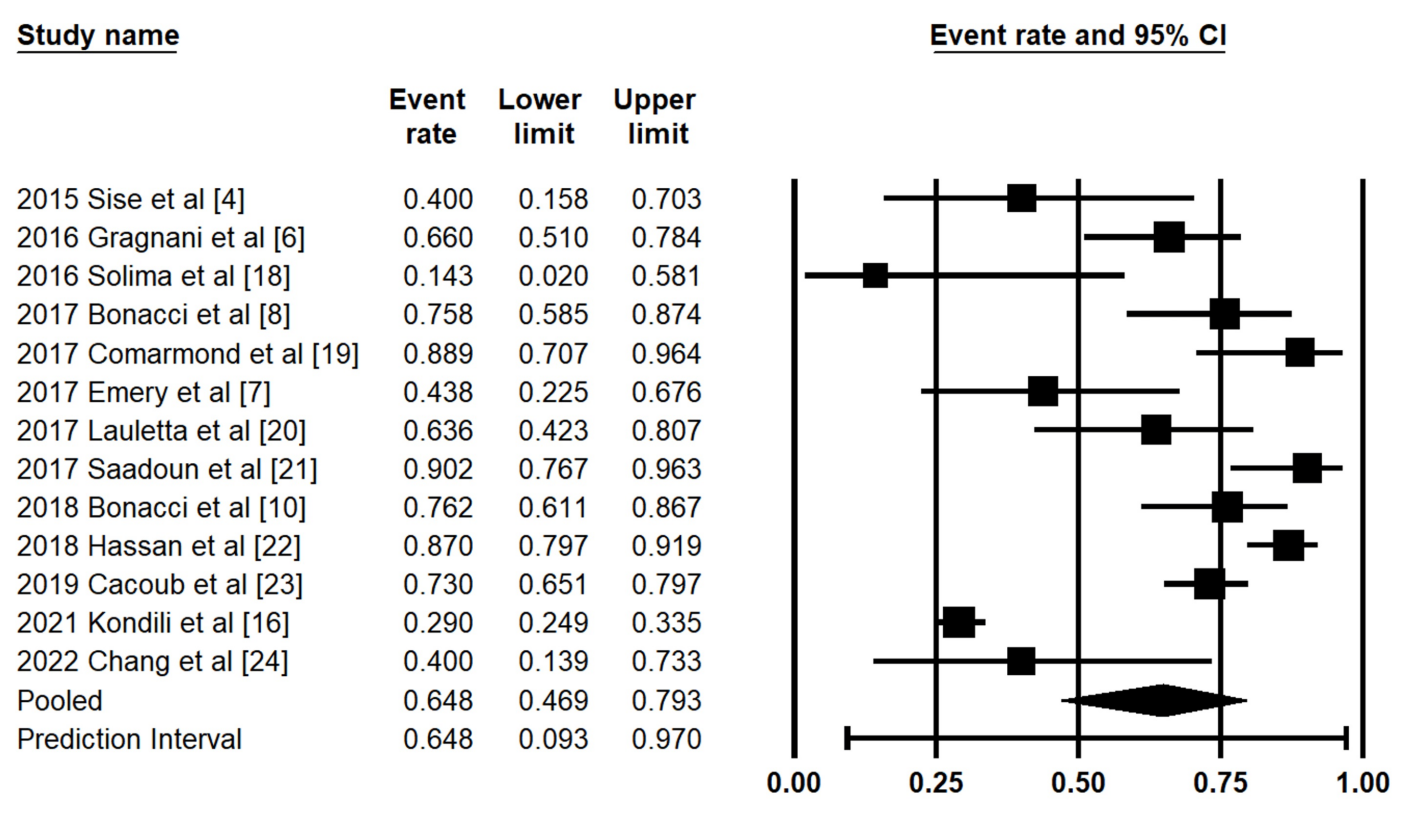
Forest plot of pooled complete clinical response at SVR12 Forest plot demonstrating pooled estimates of complete clinical response at the time of sustained virologic response at 12 weeks (SVR12). Individual study estimates are shown for cohorts reported by Sise et al. (2015) [4], Gragnani et al. (2016) [6], Solima et al. (2016) [18], Bonacci et al. (2017, 2018) [8,10], Comarmond et al. (2017) [19], Emery et al. (2017) [7], Lauletta et al. (2017) [20], Saadoun et al. (2017) [21], Hassan et al. (2018) [22], Cacoub et al. (2019) [23], Kondili et al. (2021) [16], and Chang et al. (2022) [24]. The pooled estimate and prediction interval were analyzed using a random-effects model.

A total of 13 studies reported complete clinical response outcomes at the time of SVR12. The pooled proportion of complete clinical response, conditional on achieving SVR12, was 64.8% (95% CI: 49.6%-79.3%) using a random-effects model (Figure 2). There was substantial heterogeneity across studies (I² = 94.1%).

Partial Clinical Response at SVR12

**Figure 3:**
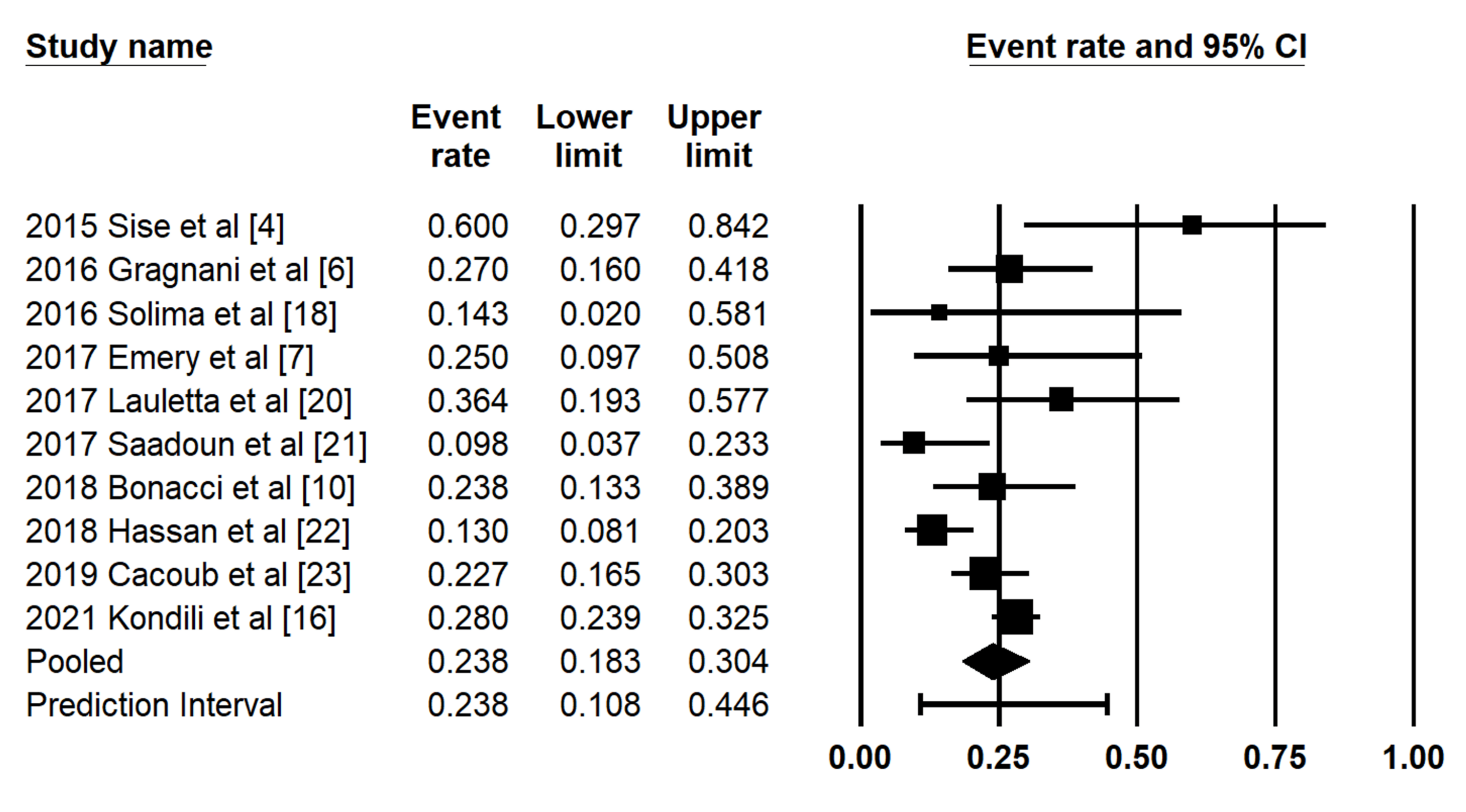
Forest plot of pooled partial clinical response at SVR12 Forest plot demonstrating pooled estimates of partial clinical response at the time of sustained virologic response at 12 weeks (SVR12). Study-level estimates are shown for cohorts reported by Sise et al. (2015) [4], Gragnani et al. (2016) [6], Solima et al. (2016) [18], Emery et al. (2017) [7], Lauletta et al. (2017) [20], Saadoun et al. (2017) [21], Bonacci et al. (2018) [10], Hassan et al. (2018) [22], Cacoub et al. (2019) [23], and Kondili et al. (2021) [16]. The pooled estimate and prediction interval were derived using a random-effects model.

A total of 10 studies reported partial clinical response outcomes at the time of SVR12. The pooled proportion of partial clinical response, conditional on achieving SVR12, was 23.8% (95% CI: 18.3%-30.4%) using a random-effects model (Figure 3). Moderate heterogeneity was observed across studies (I² = 61.2%).

Complete Immunologic Response at SVR12

**Figure 4:**
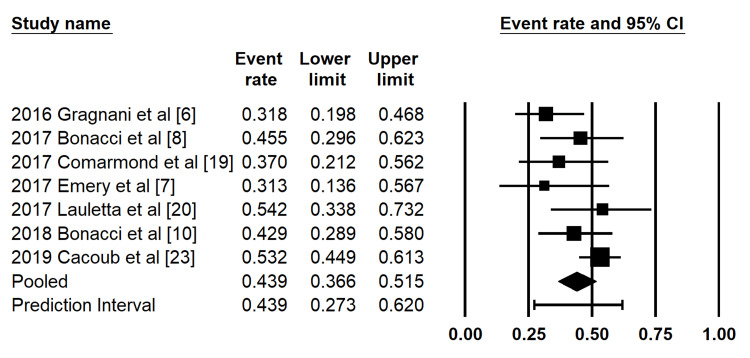
Forest plot of pooled complete immunologic response at SVR12 Forest plot demonstrating pooled estimates of complete immunologic response at the time of sustained virologic response at 12 weeks (SVR12). Individual study estimates are shown for cohorts reported by Gragnani et al. (2016) [6], Bonacci et al. (2017, 2018) [8,10], Comarmond et al. (2017) [19], Emery et al. (2017) [7], Lauletta et al. (2017) [20], and Cacoub et al. (2019) [23]. The pooled estimate and prediction interval were analyzed using a random-effects model.

A total of seven studies reported complete immunologic response outcomes at the time of SVR12. The pooled proportion of complete immunologic response, conditional on achieving SVR12, was 43.9% (95% CI: 36.6%-51.5%) using a random-effects model (Figure 4). Low to moderate heterogeneity was observed across studies (I² = 35.8%).

Discussion

In this systematic review and meta-analysis, we examined short-term clinical and immunologic outcomes conditional on achieving SVR12 among patients with symptomatic HCV-associated cryoglobulinemic vasculitis treated with DAA therapy. Among patients with confirmed viral eradication, early clinical improvement was common, with approximately two-thirds achieving a complete clinical response and an additional subset demonstrating partial response by the time of SVR12. In contrast, a complete immunologic response was observed in fewer than half of patients, highlighting a dissociation between early clinical improvement and immunologic normalization.

The favorable short-term clinical outcomes observed in this analysis are consistent with the established role of HCV as the primary antigenic driver of cryoglobulin production and vasculitic activity. Removal of chronic viral antigenic stimulation through DAA therapy appears sufficient to induce rapid attenuation of inflammatory manifestations once SVR12 is achieved [4-6]. However, the lower pooled rate of complete immunologic response suggests that immune recovery may lag behind clinical improvement. Persistence of circulating cryoglobulins, delayed normalization of complement pathways, or continued activity of expanded B-cell clones may contribute to incomplete immunologic resolution despite viral clearance [17,18,23].

Substantial heterogeneity was observed for the analysis for complete clinical response, whereas heterogeneity for immunologic response was lower. This pattern likely reflects several factors. As summarized in Table 2, definitions of complete and partial clinical response varied widely across included studies, ranging from symptom-based assessments to organ-specific or score-based definitions incorporating validated instruments, such as the Birmingham Vasculitis Activity Score [13]. In contrast, definitions of complete immunologic response were more uniform and were most commonly based on the disappearance of detectable cryoglobulins, with or without normalization of complement levels or rheumatoid factor. This greater consistency in immunologic definitions may partially explain the lower observed heterogeneity for immunologic outcomes.

In addition to the variability in definitions, included cohorts differed markedly in baseline disease severity and organ involvement (Table 1). Some studies included patients with severe manifestations, including glomerulonephritis, peripheral neuropathy, or prior need for plasma exchange, whereas others consisted predominantly of patients with cutaneous or constitutional symptoms [4,7,16]. Larger multicenter cohorts dominated by milder disease phenotypes are therefore likely to exert disproportionate influence on pooled estimates. Accordingly, the pooled proportions reported in this analysis should be interpreted as reflecting average short-term outcomes across a heterogeneous disease spectrum rather than uniform expectations for individual patients.

Concomitant immunosuppressive therapy was frequently reported across studies, including corticosteroids, rituximab, and plasma exchange; however, the timing, indication, and duration of immunosuppression varied and were inconsistently described. As a result, the independent contribution of immunosuppressive therapy to early clinical response could not be formally assessed. It is plausible that, in some patients, early clinical improvement reflects combined effects of viral eradication and immunomodulatory treatment rather than antiviral therapy alone.

Importantly, the present analysis was intentionally designed to characterize short-term outcomes at the time of SVR12, rather than long-term durability of response. Several longitudinal cohorts with extended follow-up have demonstrated that persistence or recurrence of cryoglobulinemic vasculitis may occur months to years after achievement of sustained virologic response. In a large French multicenter cohort, Cacoub and colleagues reported that although most patients experienced regression of vasculitic manifestations following viral eradication, a subset exhibited persistent or relapsing disease activity and immunologic abnormalities despite SVR, especially those with severe manifestations at baseline [23]. Subsequent long-term follow-up further demonstrated delayed or incomplete immunologic recovery and late clinical relapse after virologic cure [10].

Similar observations have been reported in Italian cohorts. Visenthini and colleagues described incomplete immunologic normalization and late relapse of cryoglobulinemic vasculitis following viral clearance [17]. Additional analyses from Italian centers have emphasized that immune system dysregulation may persist despite the elimination of HCV, supporting the concept that SVR does not uniformly translate into durable immune remission [16]. Collectively, these data indicate that early response at SVR12 does not necessarily predict long-term disease quiescence.

Taken together, these findings support a conceptual model in which viral eradication leads to rapid reduction of antigen-driven inflammation and early clinical improvement, while deeper immunologic remodeling may occur more slowly or remain incomplete in a subset of patients. Persistent immune dysregulation or clonal B-cell populations may predispose to delayed relapse, underscoring the importance of continued clinical and immunologic monitoring even after achievement of SVR12 [23].

Several limitations should be considered when interpreting these pooled results. All included studies were observational in design and therefore subject to inherent risks of selection bias and residual confounding. Definitions of clinical and immunologic response varied across studies, which likely contributed to the observed heterogeneity. In addition, the analysis was restricted to short-term outcomes assessed at SVR12 and does not address the durability of response beyond this time point. Inconsistent reporting of concomitant immunosuppressive therapy further limited the ability to evaluate its independent effects. Finally, pooled estimates reflect population-level responses and should not be directly extrapolated to patients with severe organ involvement, in whom disease course and treatment requirements may differ substantially.

Despite these limitations, this meta-analysis provides a quantitative synthesis of early post-eradication outcomes in HCV-associated cryoglobulinemic vasculitis using a conditional analytic framework. The findings indicate that, among patients who achieve SVR12 with DAA therapy, short-term clinical improvement is common, whereas complete immunologic normalization is less frequent. These results offer clinically relevant prognostic information regarding early expectations following viral eradication and complement existing studies focused on longer-term outcomes.

## Conclusions

In this systematic review and meta-analysis, we quantified short-term clinical and immunologic outcomes conditional on achieving SVR12 in patients with symptomatic HCV-associated cryoglobulinemic vasculitis treated with direct-acting antiviral therapy. Among patients with confirmed viral eradication, early clinical improvement was common, with the majority achieving complete or partial clinical response by the time of SVR12. In contrast, complete immunologic normalization occurred less frequently, indicating a dissociation between early symptomatic improvement and immunologic recovery.

These findings provide clinically relevant prognostic information regarding early expectations following SVR12 and highlight that viral eradication is often accompanied by meaningful short-term clinical benefit. However, incomplete immunologic response and evidence from long-term cohorts underscore that SVR12 does not uniformly translate into durable immune remission. Continued clinical and immunologic monitoring remains warranted beyond the short-term post-eradication period.

## Appendices

Search strategy

EMBASE

The EMBASE search was conducted using a combination of Emtree terms and free-text keywords related to hepatitis C virus infection, cryoglobulinemia or cryoglobulinemic vasculitis, and direct-acting antiviral therapy. The following search strategy was used:

('hepatitis C'/exp OR 'hepatitis C virus'/exp OR 'hepatitis C':ti,ab OR HCV:ti,ab) AND ('cryoglobulinemia'/exp OR 'cryoglobulinemic vasculitis'/exp OR cryoglobulinemia:ti,ab OR cryoglobulinaemia:ti,ab OR cryoglobulinemic:ti,ab OR cryoglobulinaemic:ti,ab) AND ('direct acting antiviral agent'/exp OR 'direct acting antiviral':ti,ab OR DAA:ti,ab OR DAAs:ti,ab OR sofosbuvir:ti,ab OR ledipasvir:ti,ab OR daclatasvir:ti,ab OR velpatasvir:ti,ab OR glecaprevir:ti,ab OR pibrentasvir:ti,ab OR elbasvir:ti,ab OR grazoprevir:ti,ab)

PubMed

The PubMed search was performed using Medical Subject Headings (MeSH) and free-text terms related to hepatitis C virus infection, cryoglobulinemia, and direct-acting antiviral therapy. The following search strategy was used:

("Hepatitis C"[Mesh] OR "Hepatitis C virus"[Mesh] OR hepatitis C[tiab] OR HCV[tiab]) AND ("Cryoglobulinemia"[Mesh] OR cryoglobulinemia[tiab] OR cryoglobulinaemia[tiab] OR cryoglobulinemic[tiab] OR cryoglobulinaemic[tiab] OR cryoglobulinemic vasculitis[tiab]) AND ("Antiviral Agents"[Mesh] OR direct acting antiviral[tiab] OR DAA[tiab] OR DAAs[tiab] OR sofosbuvir[tiab] OR ledipasvir[tiab] OR daclatasvir[tiab] OR velpatasvir[tiab] OR glecaprevir[tiab] OR pibrentasvir[tiab] OR elbasvir[tiab] OR grazoprevir[tiab])

Search Execution and Yield

The final literature search was conducted from database inception through January 31, 2026.

The PubMed search yielded 545 records, and the EMBASE search yielded 417 records.

Records retrieved from both databases were imported into EndNote and deduplicated prior to title and abstract screening.
